# Farmers’ production constraints, preferred varietal traits and perceptions on sorghum grain mold in Senegal

**DOI:** 10.1016/j.heliyon.2024.e30221

**Published:** 2024-04-26

**Authors:** Cyril Diatta, Thierry Klanvi Tovignan, Bassirou Sine, Beatrice Elohor Ifie, Jacques Martin Faye, Elisabeth Diatta-Holgate, Fatou Anna Sylla, Souleymane Bodian, Ousmane Aidara, Eric Yirenkyi Danquah, Samuel Kwame Offei, Ndiaga Cisse

**Affiliations:** a Institut Sénégalais de Recherches Agricoles (ISRA), Centre d’Etude Régional pour l’Amélioration de l’Adaptation à la Sécheresse (CERAAS), BP 3320, Thiès Escales, Senegal; b Institut Sénégalais de Recherches Agricoles (ISRA), Centre National de Recherches Agronomiques (CNRA) de Bambey, BP 53, Bambey, Senegal; c Département de Génétique et des Biotechnologies, Faculté des Sciences et Techniques (FAST), Université d’Abomey-Calavi (UAC), BP 1947, Cotonou, Benin; d West Africa Centre for Crop Improvement, University of Ghana, PMB LG 30, Legon, Ghana; e Centre Haïtien d’Innovation en Biotechnologies et pour une Agriculture Soutenable (CHIBAS), Faculté des Sciences de l’Agriculture et de l’Environnement, Université Quisqueya, 218 Ave Jean-Paul II, Port-au-Prince 6110, Haiti

**Keywords:** Sorghum constraints, Participatory rural appraisal, Focus group discussion, Farmers' preferences, Product profile, Senegal

## Abstract

Improving sorghum adoption rates by developing adapted varieties that meet end-user preferences is a major challenge in West Africa. In this study, a participatory rural appraisal was undertaken to identify the main sorghum production constraints, farmers’ preferred variety traits and their perceptions on sorghum grain mold. The study was conducted in four representative rural communities located in the main sorghum producing area of Senegal. A total of 260 farmers were interviewed and data were collected through focus group discussions and individual questionnaires. Our results indicated that Striga, insects, poor soil fertility and drought are the major sorghum producing constraints in Senegal. Grain mold was identified as the second most important sorghum disease after the damping-off. Discoloration on grain surface was the most important criteria farmers used to recognize the disease. The most important sorghum traits farmers desired in improved varieties are medium to short plant maturity cycle, medium plant height, large open or semi-compact panicle, big and white grain, and adaptation to local growing conditions. The results showed that the sorghum cropping system is dominated by male farmers who mainly grow local landraces. These results will provide updated recommendations for the breeding products profile to meet end-user preferences in Senegal.

## 1 Introduction

Sorghum is a very important cereal crop in the arid and semi-arid zones of Africa, Asia, and the Americas. It ranks second in West Africa among the most cultivated cereal crop, serving as the primary source of energy, protein, vitamins, and minerals for many households [1]. In Senegal, sorghum is widely grown and represents the second most grown cereal crop during the rainy season after pearl millet [2]. It is also grown along the Senegal River valley during the water flooding recession. Rainfed sorghum is produced in the agro-ecological zones of the groundnut basin, Eastern-Senegal and Casamance. In 2020, in Senegal, sorghum was grown on 238,833,502 ha of land with a production of 270,168 tons, giving an average grain yield of 1.131 tons ha^−1^ [3], which is far below the potential of 7–11 tons ha^−1^ [4]. The five most important sorghum producing regions in Senegal are: Kaffrine (96,237 tons), Tambacounda (62,457 tons), Kolda (56,463 tons), Sedhiou (47,712 tons) and Kaolack (39,733 tons) [2,3].

To increase sorghum grain yield, over 40 cultivars have been released in sub-Saharan Africa [5]. However, only a few of them have been adopted by farmers. A very low rate of adoption has been reported in most West African countries [6,7]. In the 1980's when most of these varieties were released, their rate of adoption was at 5 % [8]. Up to the present, the level of adoption has remained low in many countries. For instance, 5 % was reported in Burkina Faso**,** 17–30 % in Mali, 24–38 % for the variety S35 in Chad, 25 % in Nigeria, and 10 % in Senegal [5,[9], [10], [11], [12]]. According to Witcombe et al. [13], the low level of adoption of improved cultivars in the developing countries can be explained by the fact that farmers in marginal areas have not been exposed to acceptable alternatives compared to their landraces. Improved varieties are more demanding with respect to the sowing date (non-photoperiodic), fertilizer, good agronomic practices and usually the quality of grain are less appropriate for conservation and preparation of local diets [8]. Kaliba et al. [14], using data collected from 822 farmers' households in Tanzania, showed that the lack of basic resources (land, capital, financial support from government) are among the factors slowing down the producers willing to move towards the use of the improved varieties. These authors suggested raising awareness among farmers through communication and in particular by organizing special events like “Field Days” to showcase the performance of the improved varieties in field plots. In addition, in Burkina Faso, Barry et al. [15], in a study focusing on 300 farmers, pointed out easy access to credit and the availability of improved varieties as the means to increase the adoption rate of improved varieties. However, Sanou et al. [12] thought that the breeders are still have an incomplete understanding of why farmers choose the varieties they grow.

In Senegal, considerable progress has been made by the Senegalese Institute of Agricultural Research (ISRA) in improving genetic material for sorghum yield and grain quality. These efforts, in 2011 and 2015, led to the release of six new tannin-free varieties, namely Nguinthe, Faourou, Nganda, Darou, Payenne, and Golobe. These varieties yield up to 4 tons ha^−1^ and were highly appreciated by farmers for the quality of their grain. However, some complaints from farmers about the sensitivity to grain mold leading to poor germination have been reported. In fact, these varieties bred for earliness, in a prolonged growing season, matured before the end of the rainy season and became infected by grain mold. This disease is one of the main constraints to the adoption of improved short- and medium-duration photo-insensitive sorghum in Senegal [16,17].

Therefore, alternative approaches to increasing the level of adoption of improved varieties by small-scale farmers are urgently needed in Senegal. One of the most widely used methods of identifying farmers’ preferences for a cultivar is through participatory rural appraisal (PRA). According to Witcombe et al. [18], a successful PRA provides the information needed to specify the characteristics required in a new variety, the physical environment, the existing varietal diversity, the size of the market, and the desired traits. In Senegal, few studies have been conducted to provide valuable information that can help the breeding program in identifying or setting their priorities.

The present study aimed at identifying (i) the main sorghum production constraints and (ii) the farmers' trait preferences for the adoption of new sorghum varieties. It also sought to understand (iii) the farmers’ perception on sorghum grain mold in the main sorghum production areas of Senegal.

## 2 Materials and methods

### 2.1 Description of the study area

**Fig. 1:**
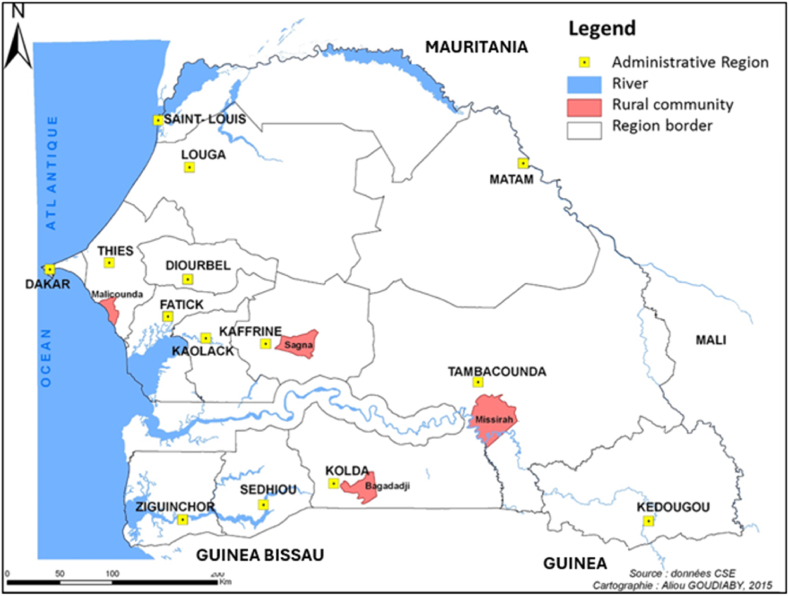
Location of the four rural communities (in red) in the Senegal map. This map was drawn using Quantum Geographic Information System (QGIS, Version 2.14.8).

The Republic of Senegal is administratively subdivided and decreasingly in regions, departments, districts, rural communities, and villages. A participatory rural appraisal (PRA) study was conducted in four rural communities (Bagadadji, Malicounda, Missirah and Sagna) located in the main sorghum cultivation zone in Senegal (Fig. 1). The sites were selected based on the latest agricultural statistics available at the National Agency of Statistics and Demography (ANSD) [19]. The selections were based on grain production and the relative importance of sorghum in the livelihoods of small-scale farmers. These four rural communities are distributed in three different agro-ecological zones. The first two districts Sagna in Kaffrine region and Malicounda in Thies region, are in the central-south and central-north of the Groundnut basin zone, respectively. In these areas, sorghum is generally sown in a sandy-clay soil locally called *Dior-deck*. The climate is sudano-sahelian and the rainfall varies between 500 and 800 mm from July to October. The third site is the rural community of Missirah in Tambacounda region situated in eastern Senegal. The soils in this zone are predominantly clay (*Deck*) and the climate is sudanian with a rainfall between 800 and 1000 mm from June to November. The fourth site is the rural community of Bagadadji in Kolda region. This region belongs to the agro-ecological zone of Casamance, in southern Senegal. In Casamance, sorghum is generally grown in clay soils (*Deck*). The climate of this region is sub-Guinean with a rainfall above 1000 mm per year from June to November.

### 2.2 Sampling of respondents

In each rural community, five to eight villages were selected, based on importance of sorghum production and their accessibility. In each village with the help of its head, only farmers engaged in sorghum cultivation were selected and interviewed. Additionally, three focus group discussions were held in the three biggest villages of each rural community. In Malicounda and Sagna, the majority of farmers involved were members of the local producers’ cooperative, which is affiliated to RESOPP (*Réseau des Organizations Paysannes et Pastorales du Sénégal*) and the producers cooperative of Saloum (COPROSA) that belong to ASPRODEP (*Association Sénégalaise pour la promotion du développement à la Base*). However, in Bagadadji and Missirah, only farmers who had grown sorghum the previous year were selected.

### 2.3 Data collection

The data were collected through both a formal household survey and an informal or Participatory Rural Appraisal (PRA). The PRA involved three focus group discussions organized in the three biggest villages of each rural community. A total of 12 focus group discussions were organized in all the four rural communities. Each focus group comprised 15 farmers (10 males and 5 females) making a total of 45 farmers per site and 180 farmers in all the four rural communities. Different topics such as major crops grown, soil types, sources of sorghum seeds, sorghum planting and harvesting time, sorghum production, constraints limiting sorghum production, farmers’ perception on grain mold and preferred sorghum traits were used to guide the discussion. The focus group discussion was followed up by formal surveys using semi-structured questionnaires. The formal survey involved individual interviews with 65 farmers per site, making a total of 260 farmers from the four rural communities covered by this study. This enabled individual farmers to express their own opinions without any inﬂuence from the community. During the survey, practical demonstrations and drawings were used to illustrate difficult points. For instance, the team utilized visual aids such as pictures to assist farmers in identifying various diseases, including grain mold, ergot, long smut, and foliar diseases. Sorghum stems, panicles, seeds with different characteristics were also exposed to help farmers in selecting their preferred traits.

### 2.4 Data analysis

Data collected from focus group discussions and individual questionnaires were coded and analyzed using the Statistical Package for Social Sciences (SPSS) version 19. Descriptive statistics, *t*-test and chi-square tests were performed using the same computer package.

## 3 Results

### 3.1 Demographic and socio-economic background

Of the 260 farmers interviewed in the rural communities of Bagadadji, Malicounda, Missirah and Sagna, 87.7 % were male and 12.3 % were female (Table 1). Most of the active farmers (51.2 %) were between 25 and 50 years old with only 3.8 % less than 25 years old and 17 % more than 75 years old. Farmers’ ethnic group differed significantly (P < 0.0001) between locations. In Bagadadji, all the farmers interviewed belonged to the *Foulany* ethnic group while in Malicounda and Sagna most of them belonged to the *Serere* ethnic group and the *Wolof* ethnic group, respectively. A great diversity of ethnic groups was noted in Missirah rural community. The educational status of farmers differed significantly (P < 0001) between rural communities. Of the total respondents, 41.5 % had attended Koranic school and 15.8 %, adult literacy, those who were trained to write and read one of the local languages and using it in their daily lives. Only 11.2 % of the farmers were educated in French at the primary level, 6.5 % up to secondary level and 23.8 % without any education (Table 1).

**Table 1:** Household characteristics in each rural community.

Trait	Modalities	Rural Communities				Total	Percent	Chi-Square		
		Bagadadji	Malicounda	Missirah	Sagna			Value	Df	P-Value
**Gender of household head**	**Male**	56	58	56	58	228	87.7	0.6	3	0.903
	**Female**	9	7	9	7	32	12.3			
	**Total**	65	65	65	65	260	100.0			
**Age of household head (in years)**	**< 25**	2	1	3	4	10	3.8	20.8	9	0.014
	**25**–**50**	44	24	29	36	133	51.2			
	**51**–**75**	18	33	26	23	100	38.5			
	**> 75**	1	7	7	2	17	6.5			
	**Total**	65	65	65	65	260	100.0			
**Ethnic group of household head**	**Diahanke**	0	0	15	0	15	5.8	542.3	27	0.000
	**Foulany**	65	4	25	1	95	36.5			
	**Sarakhole**	0	1	3	0	4	1.5			
	**Serere**	0	53	2	0	55	21.2			
	**Soninke**	0	0	8	0	8	3.1			
	**Wolof**	0	4	0	63	67	25.8			
	**Toucouleur**	0	1	0	1	2	0.8			
	**Bambara**	0	1	8	0	9	3.5			
	**Manding**	0	0	4	0	4	1.5			
	**Nar**	0	1	0	0	1	0.4			
	**Total**	65	65	65	65	260	100.0			
**Level of education**	**Illiterate**	10	38	13	1	62	23.8	114.6	15	0.000
	**Adult literacy**	20	0	7	14	41	15.8			
	**Koranic study**	22	10	31	45	108	41.5			
	**Primary**	11	6	7	5	29	11.2			
	**Secondary**	2	10	5	0	17	6.5			
	**Tertiary**	0	1	2	0	3	1.2			
	**Total**	65	65	65	65	260	100.0			

### 3.2 Cropping systems

#### 3.2.1 Characterization of farmers’ production system in the four rural communities

The majority of the farmers had at least two to four fields (60.5 %) with only 15.4 % having one field (Table 2). Sorghum was primarily grown as a pure crop by 97.3 % of farmers. Some cases of association with groundnut and cowpea were noted, but still very low. The type of variety grown differed significantly (P < 0.001) from one rural community to another. Generally, 71.5 % of farmers used local landraces, while only 10.8 % used improved varieties. Some of them preferred using both local landraces and improved varieties (17.5 %). The number of varieties grown per farmer varied significantly (P < 0.0001) from one locality to another. Overall, between one and six varieties were grown per farmer with the majority cultivating only one variety (61.9 %). Most farmers (69.6 %) cultivated sorghum on one to 2 ha and less than 16.2 % cultivated on less than 1 ha. The largest acreage per farm was recorded at Malicounda where more than 6 ha were sown with sorghum. The tractions systems used in land preparation differed significantly (P < 0.0001) among rural communities.

**Table 2:** Characterization of farmer's production system.

Traits	Responses	Rural communities				Total	Percent	Chi-square		
		Bagadadji	Malicounda	Missirah	Sagna			Value	Df	P. Value
**Agronomic practice**	**Monoculture**	64	64	65	60	253	97.3	8.7	3	0.034
	**Double cropping**	1	1	0	5	7	2.7			
	**Total**	65	65	65	65	260	100.0			
**Type of varieties**	**Local landraces**	65	21	63	37	186	71.5	157.9	6	0.000
	**Improved**	0	28	0	0	28	10.8			
	**Improved and Local landraces**	0	16	2	28	46	17.7			
	**Total**	65	65	65	65	260	100.0			
**Number of varieties grown**	**One**	50	26	53	32	161	61.9	86.7	15	0.000
	**Two**	15	11	12	26	64	24.6			
	**Three**	0	18	0	7	25	9.6			
	**Four**	0	6	0	0	6	2.3			
	**Five**	0	3	0	0	3	1.2			
	**Six**	0	1	0	0	1	0.4			
	**Total**	65	65	65	65	260	100.0			

#### 3.2.2 Sorghum landraces and varieties grown by farmers in each rural community

**Fig. 2:**
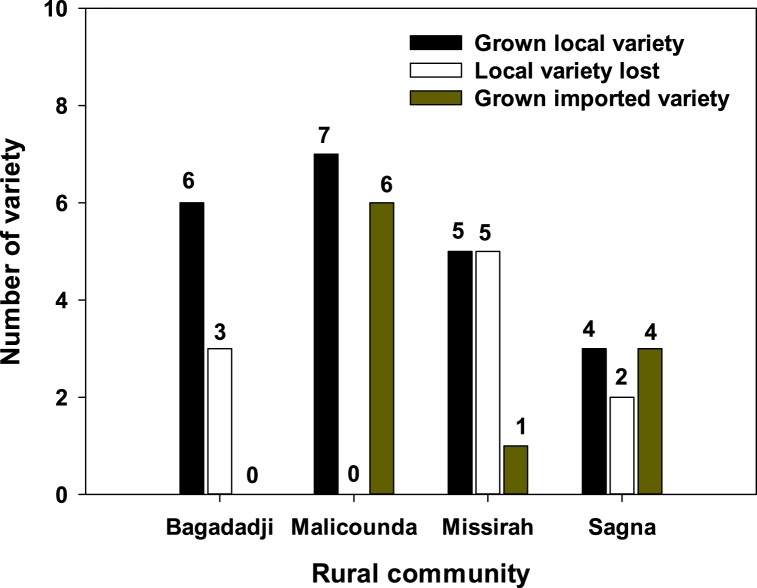
Number of improved and local sorghum varieties grown or lost by farmers in each rural community.

Overall, 33 landraces and 6 improved varieties were listed by farmers in the four rural communities, during a formal and informal survey (Fig. 2). The 6 major local landraces preferred by farmers at Bagadadji were Mbayery danery, Mbayery mbodery, Mbayery mobal, and Teigne. At Malicounda, the three local varieties preferred by farmers are Bassi mbodiene, Tigne, and Fellah. At Missirah the mostly used were Thiamery, Niodjé, Niodjé kounkounfi, Nianikel and Nianykou. At Sagna, these were Cogossane, Bassi and Tellanior. A relatively important number of landraces (10) were no longer cultivated by farmers. These landraces were Kintiry, Niany kinty and Samba diabo at Bagadadji; kandé djé, Kinto djéma, kinto woulé, Mbadar cimakan and Samba Traoré at Missirah; and Yabé and Yamar at Sagna. The reasons given by farmers for leaving these local varieties are numerous. For instance, they claimed that the use of very late maturing local landraces is very risky because they are frequently subjected to terminal drought, bird attacks and animal intrusion. With regard to the very early maturing local varieties, farmers think such varieties usually mature before the end of the rainy season, leaving them vulnerable to grain mold and bird attacks. Of the 10 improved varieties released by the sorghum breeding program of ISRA between 1980 and 2011, only six (6) were being used by farmers in the four rural communities. Malicounda was the most important locality where improved varieties were adopted by farmers (Fig. 2, Table S1). The second most important rural community with cultivated improved varieties was Sagna. In this locality, farmers cultivated both landraces and improved varieties. The new varieties Nguinthe, Faourou and Darou released in 2011 were the most grown by farmers in the rural communities of Malicounda and Sagna. Nevertheless, the improved varieties CE151-262 and CE180-33 released in 1981 were still grown by farmers except at Bagadadji.

#### 3.2.3 Post-harvest management of sorghum

Post-harvest management of sorghum differed significantly (P < 0.0001) from one rural community to another (Table 3). After harvesting, 63.8 % of the farmers at Bagadadji, Missirah and Sagna dry the panicles in the field while at Malicounda, farmers preferred drying their harvest at home. The duration of panicles drying varied from one to four weeks and this is dependent on the time used to harvest their groundnut field. Grains are mostly stored (73.1 %) as seeds at Malicounda, Missirah and Sagna, while at Bagadadji, it was the entire panicles (26.9 %). After drying, 72 % of the farmers at Malicounda, Missirah and Sagna threshed panicles, put grains into nylon bags (rice bags) and stored in their house (44.2 %) or their warehouses (41.5 %). However, at Bagadadji, panicles were tied in bundles (22.3 %) and kept in a hut near the house.

**Table 3:** Management of the harvest in the study area.

Traits	Responses	Rural communities				Total	Percent	Chi-Square		
		Bagadadji	Malicounda	Missirah	Sagna			Value	Df	P. Value
Place of drying	Field	46	7	54	59	166	63.8	111.50	3	0.000
	House	19	58	11	6	94	36.2			
	Total	65	65	65	65	260	100			
Time of drying	Less than a week	17	4	3	3	27	10.4	58.61	15	0.000
	One week	13	11	18	12	54	20.8			
	Two weeks	6	13	19	22	60	23.1			
	Three weeks	5	2	13	10	30	11.5			
	One Month	16	23	11	14	64	24.6			
	More than a month	8	12	1	4	25	9.6			
	Total	65	65	65	65	260	100			
Place of storage	Warehouse	6	9	40	53	108	41.5	205.99	9	0.000
	Inside the house	23	55	25	12	115	44.2			
	Hut near house	36	0	0	0	36	13.8			
	Roof of the house	0	1	0	0	1	0.4			
	Total	65	65	65	65	260	100			
Form of seed storage	Grain	6	62	57	65	190	73.1	215.77	6	0.000
	Bulk panicles	1	3	5	0	9	3.5			
	Panicles tied in bundles	58	0	3	0	61	23.5			
	Total	65	65	65	65	260	100			

#### 3.2.4 Seed source, planting and harvesting periods

The majority of farmers interviewed (66.9 %) in the four rural communities indicated that the seeds they planted came from their own farm (Table 4). Only a few farmers (12.3 %) in Malicounda and Sagna used seeds from official seed production services. At Malicounda, farmers obtained sorghum seeds from the Cooperative of the locality (COPAM) and in Sagna, seeds were obtained from producers’ cooperative of Saloum (COPROSA). A limited number of farmers (5 %) obtained seeds from the market. According to these farmers, they did not have any alternative sources even though they were aware that seeds bought from the market are often not good because of their low rate of germination. The planting period in the different rural communities was dependent on the onset of rainfall (P < 0.0001). In Bagadadji, Missirah and Sagna, where the rainy season starts early, most of the farmers (78 %) plant their sorghum between June and July. In Malicounda, where the rainy season starts late, the main planting period of sorghum was August. However, late maturing landraces were planted before rainfall starts (2.3 %) in order to benefit from the maximum amount of rain. Only improved varieties are generally sown in August in most of the villages of the rural community of Malicounda in order to avoid grain molding. The period of harvesting, in the four rural communities, ranges from October to January. Most of the farmers (69.6 %) that plant early maturing varieties harvest their field before 15th of November. The other farmers (25.8 %) harvest their field between mid-November and mid-December. Only 4.6 % of the farmers harvest the late maturing landraces after 15th of December.

**Table 4:** Seed source and farmers planting and harvesting period in the different rural communities.

Traits	Responses	Rural Communities				Total	Percent	Chi-Square		
		Bagadadji	Malicounda	Missirah	Sagna			Value	Df	P. Value
**Seed source**	Own farm	60	17	63	34	174	66.9	188.87	21	0.000
	Market	2	5	1	5	13	5.0			
	Cooperative	0	32	0	0	32	12.3			
	Relative	3	2	0	0	5	1.9			
	Own farm & Cooperative	0	9	1	23	33	12.7			
	Own farm & Market	0	0	0	2	2	0.8			
	Market & Cooperative	0	0	0	1	1	0.4			
	**Total**	**65**	**65**	**65**	**65**	**260**	**100**			
**Period of sowing**	Before the rain	0	6	0	0	6	2.3	162.32	12	0.000
	Mid-June to Mid-July	65	16	65	59	205	78.8			
	Mid-July to End-July	0	17	0	6	23	8.8			
	Start-August to Mid-August	0	21	0	0	21	8.1			
	Mid-August to End-August	0	5	0	0	5	1.9			
	**Total**	**65**	**65**	**65**	**65**	**260**	**100**			
**Period of harvesting**	Mid-October to Mid-November	62	15	58	46	181	69.6	105.63	6	0.000
	Mid-November to Mid-December	3	42	3	19	67	25.8			
	Mid-December to Mid-January	0	8	4	0	12	4.6			
	**Total**	**65**	**65**	**65**	**65**	**260**	**100**			

### 3.3 Use of fertilizer and pesticide

The type of fertilizer used by farmers differed significantly (P < 0.0001) among rural communities. Of the total 260 farmers interviewed, only 91 (35 %) used mineral fertilizers in their fields (Table 5). Most of these farmers lived in Sagna where sorghum is generally grown in poor sandy clay soil. According to farmers, with the low soil fertility, the use of fertilizer is key for sorghum production in this area. However, 70.3 % of the farmers applying mineral fertilizers in their field claimed that the cost of NPK (Nitrogen, Phosphorus and Potassium) fertilizers and Urea (46 %) is very expensive. This explained the non-compliance to the recommendation of ISRA (Senegalese Institute of Agricultural Research) which prescribes 150 kg ha^−1^ for NPK and 100 kg ha^−1^ for Urea (46 %) in two applications of 50 kg ha^−1^ each.

**Table 5:** Farmers' utilization of fertilizers and pesticides, alongside their perspectives on associated costs.

Traits	Responses	Rural Communities				Total	Percent	Chi-Square		
		Bagadadji	Malicounda	Missirah	Sagna			Value	Df	P. Value
Use of mineral fertilizer	Yes	11	15	14	51	91	35.0	72.54	3	0.000
	No	54	50	51	14	169	65.0			
	Total	65	65	65	65	260	100.0			
Perspective on the price of fertilizer	Affordable	1	2	3	11	17	18.7	4.48	6	0.612
	Average	2	3	0	5	10	11.0			
	Expensive	8	10	11	35	64	70.3			
	Total	11	15	14	51	91	100.0			
Use of pesticides	Yes	3	37	26	55	121	46.5	87.73	3	0.000
	No	62	28	39	10	139	53.5			
	Total	65	65	65	65	260	100.0			
Perspectives on the price of pesticides	Affordable	0	10	4	6	20	17	14.18	6	0.028
	Average	1	12	2	11	26	22.0			
	Expensive	2	13	19	38	72	61.0			
	Total	3	35	25	55	118	100.0			

The use of pesticides was uncommon among farmers in the four rural communities (P < 0.0001). Most of the farmers (54.6 %) especially those living in Bagadadji and Missirah did not apply pesticide at any time in the farming process. Only 45.4 % of the farmers, especially those in Malicounda and Sagna treated their seeds with an insecticide/fungicide such as, Granox and Sahal before planting. Among the farmers that apply fertilizer, 61 % consider that the cost of pesticides is prohibitively high.

### 3.4 Use and importance of sorghum among farmers

**Fig. 3:**
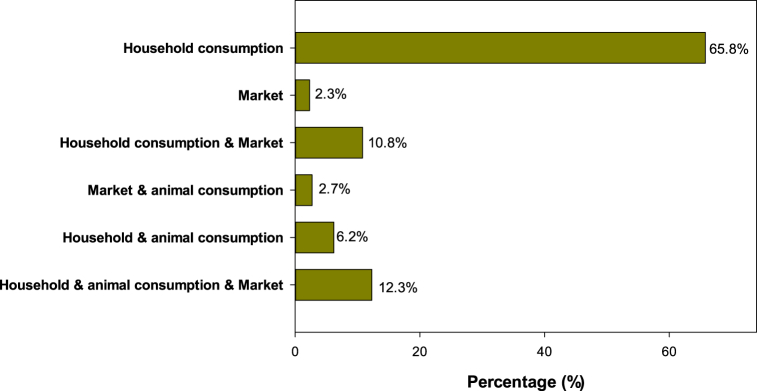
Different uses of sorghum grain by farmers.

Fig. 3 shows that 65.8 % of the farmers in the four rural communities grow sorghum only for household consumption. Only 12.3 % of farmers use sorghum for household consumption, animal feeding and marketing. The farmers using sorghum for household consumption and market represented 10.8 %. Very few farmers (2.3 %), mainly from Malicounda and Sagna, grew sorghum solely for marketing. In Bagadadji and Missirah where local landraces are mostly grown, sorghum is mainly used for household consumption. In Malicounda and Sagna where farmers grew both local landraces and improved varieties, the grains are mainly used for household consumption while the surplus are sold and/or used for animal feed. According to farmers from Malicounda and Sagna, the price of sorghum grain per kilogram (200–300 F CFA) was higher than that of pearl millet (150–200 F CFA).

**Fig. 4:**
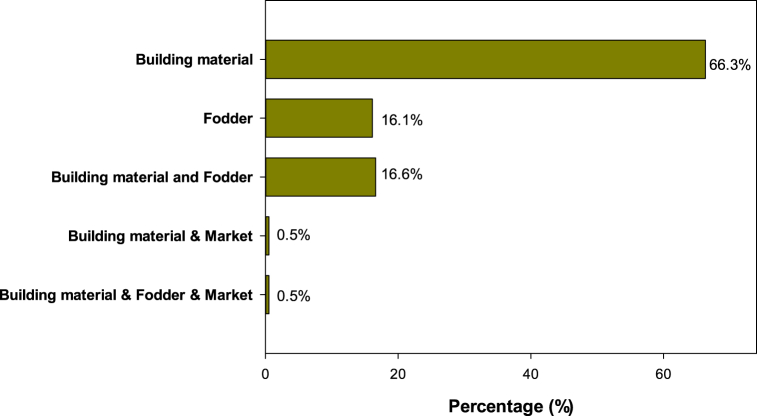
Use of sorghum straws by farmers in the four rural communities.

Overall, in the four rural communities, sorghum straws were primarily used as building material (66.3 %), fodder (16.1 %) and both building material and fodder (16.6 %) (Fig. 4). Most of the farmers using the plant as forage or building material and forage come from Malicounda and Sagna where dual purpose varieties are released. In Bagadadji and Missirah, the most humid rural communities with rainfall ranging from 800 to 1200 mm, farmers reported that animal feed was not a significant concern. Consequently, a small quantity of straw was harvested and used as building material, while the remainder was left in the field.

### 3.5 Relative ranking of sorghum production constraints

The relative ranking of sorghum production constraints differed significantly (P < 0.001) between the four rural communities (Table 6). Overall, the most important sorghum production constraints were striga, insects, low soil fertility, land access, lack of rain, birds, animal intrusion, diseases, and agricultural inputs. Nevertheless, at Malicounda, farmers ranked bird attacks as the main constraint followed by insects and animal intrusion. While in Sagna, striga was considered the most important constraint, followed by land access and lack of rain respectively. At Bagadadji and Missirah, striga and low soil fertility were the two most important constraints. Farmers did not consider diseases as important constraints impacting sorghum production in their area.

**Table 6:** Relative ranking of major sorghum production constraints in each of the four Rural Communities.

Constraints	Rural Communities								Overall Mean	Std. Deviation	Rank	P. Value
	Bagadadji		Malicounda		Missirah		Sagna					
	Mean	Rank	Mean	Rank	Mean	Rank	Mean	Rank				
**Striga**	1.41	1	4.57	9	1.43	1	1.91	1	2.33	1.34	1	0.021
**Insects**	3.46	4	1.80	2	3.04	3	3.25	4	2.89	0.64	2	0.001
**Low soil fertility**	2.43	2	3.67	8	2.37	2	3.36	5	2.96	0.59	3	0.000
**Land Access**	2.50	3	3.43	5	3.50	5	2.75	2	3.05	0.43	4	0.000
**Lack of Rain**	3.46	4	3.12	4	3.26	4	3.08	3	3.23	0.15	5	0.000
**Birds**	4.50	8	1.76	1	3.84	7	4.07	6	3.54	1.07	6	0.002
**Animal intrusion**	4.24	7	2.93	3	3.60	6	5.41	9	4.05	0.92	7	0.001
**Diseases**	4.17	6	3.51	6	3.92	8	5.17	8	4.19	0.62	8	0.000
**Agricultural input**	4.60	9	3.57	7	4.00	9	4.91	7	4.27	0.52	9	0.000

### 3.6 Relative ranking of the most important sorghum diseases

The most important diseases frequently observed by farmers in their fields were damping off, grain mold, smut, stem diseases, and foliar diseases (Table 7). Among these diseases, damping off was ranked first, followed by grain mold and long smut. In the rural community of Malicounda, foliar diseases and stem diseases were not considered as constraints, whereas in Missirah, only foliar diseases were not impacting sorghum production.

**Table 7:** Relative ranking of the most important sorghum diseases listed by farmers in the different sorghum growing area.

Disease	Rural Communities								Overall Mean	Std. Dev	Rank	P. Value
	Bagadadji		Malicounda		Missirah		Sagna					
	Mean	Rank	Mean	Rank	Mean	Rank	Mean	Rank				
**Damping off**	1.59	1	1.71	2	1.50	1	1.63	1	1.66	0.37	1	0.00
**Grain Mold**	1.84	2	1.71	2	1.93	2	1.95	3	1.86	0.10	2	0.00
**Long Smut**	2.12	4	1.57	1	1.98	3	1.84	2	1.88	0.20	3	0.00
**Stem Diseases**	2.00	3	**-**	**-**	2.10	4	3.56	4	2.88	0.73	4	0.01
**Foliar Diseases**	2.50	5	**-**	**-**	**-**	**-**	3.58	5	3.39	0.58	5	0.01

### 3.7 Farmer's perceptions on sorghum grain mold

Of the 260 farmers interviewed, 71 % encountered grain mold disease in their fields (Table 8). This ranged from about 51 % in Missirah to 86 % in Sagna. The discoloration on grain surface was the most important criterion used by farmers to identify the disease. The most important color cited by farmers in various rural communities was the grains turning black (70 %), black and pink (18 %), and pink (10 %). Most of these farmers (76 %) reported that the last rains usually coincided with the maturity of their sorghum. Farmers attributed these discolorations to the rain (64 %), high moisture (16 %) and insects (8 %). Some of them (12 %) reported that they did not know the reason for the discolorations. Farmers usually faced low germination rates at planting (Table S2). Lack of seed germination was reported by 90 % of the interviewed farmers. Various reasons were given to explain the low germination rate observed in their sorghum fields. Among these, were low-quality seeds (23 %), insects (13 %), lack of rain (11 %), poor soil fertility (11 %), poor rainfall and insects (11 %), and their interactions were the most cited by farmers.

**Table 8:** Characterization of sorghum grain mold by farmers in different rural communities.

Traits	Responses	Rural communities				Total	Percent	Chi-Square		
		Bagadadji	Malicounda	Missirah	Sagna			Value	Df	P.Value
Rain at maturity	Yes	53	49	41	54	197	76	8.8	3	0.032
	No	12	16	24	11	63	24			
	**Total**	**65**	**65**	**65**	**65**	**260**	**100**			
Grain mold problems	Yes	47	49	33	56	185	71	20.9	3	0.000
	No	18	16	32	9	75	29			
	**Total**	**65**	**65**	**65**	**65**	**260**	**100**			
Color observed on the grain surface	Black	37	44	26	22	129	70	46.0	12	0.000
	Pink	1	1	3	13	18	10			
	White	1	1	0	1	3	2			
	Black& Pink	7	2	4	20	33	18			
	Black & White	1	1	0	0	2	1			
	**Total**	**47**	**49**	**33**	**56**	**185**	**100**			
Reasons for discoloration	Rain	23	21	25	45	114	64	54.1	21	0.000
	Insect	2	3	1	2	8	4			
	Bad drying	0	0	0	2	2	1			
	Early harvest	1	0	0	0	1	1			
	Grain mold	2	0	0	1	3	2			
	High moisture	11	18	0	0	29	16			
	Dew	1	0	0	0	1	1			
	Unknown	7	4	5	5	21	12			
	**Total**	**47**	**46**	**31**	**55**	**179**	**100**			
Germination problems	Yes	55	62	56	61	234	90	5.6	3	0.136
	No	10	3	7	4	24	9			
	**Total**	**65**	**65**	**65**	**65**	**260**	**100**			

### 3.8 Farmers’ preferred plant characteristics for improved sorghum varieties

Seven important morphological descriptors were used in order to assess farmers’ preferred plant characteristics for improved sorghum varieties (Table 9). Except for panicle length and grain color, farmers desired plant morphological traits in an improved sorghum variety, varied from one rural community to another (P < 0.0001). Tan plant color rather than anthocyanin (20.4 %) was preferred by 79.6 % of the farmers. Farmers at Bagadadji, Missirah, and Sagna preferred varieties with medium (61.2 %) and tall (24.6 %) plants, whereas at Malicounda, short (14.2 %) and medium plant height were chosen. Semi-compact (44.6 %) and loose panicle (34.2 %) were particularly preferred by farmers. Loose panicles were mainly chosen by farmers from Bagadadji and Missirah, where the guinea race represents the majority of sorghum landraces grown. Whereas at Malicounda and Sagna where improved varieties were adopted, farmers preferred mostly semi-compact panicles. Eighty-three point eight percent (83.8 %) of the farmers desired long panicles rather than medium (15.4 %) or small ones, respectively. Medium (50.8 %) and large (39.2 %) grain size were the two most important grain criteria that farmers wanted to have in improved sorghum varieties. Almost, all farmers (98.8 %) manifested their high interest in white grain sorghum varieties. Only two farmers with diabetes had red grains as their favorites. Preferences of farmers for duration to plant maturity differ from one locality to another (P < 0.000). Hence, 67.7 % of farmers preferred medium maturity, while 30.8 % preferred short maturity sorghum. These farmers reported that growing late maturing varieties was very risky, because they are subject to terminal drought, bird, and animal attacks.

**Table 9:** Farmers preferred plant characteristics in improved sorghum variety in the four rural communities.

Traits	Responses	Rural Communities				Total	Percent	Chi-Square		
		Bagadadji	Malicounda	Missirah	Sagna			Value	Df	P. Value
**Plant color**	Tan	57	62	48	40	207	79.6	27.0	3	0.000
	Anthocyanin	8	3	17	25	53	20.4			
	**Total**	**65**	**65**	**65**	**65**	**260**	**100**			
**Plant height**	Short	1	33	0	3	37	14.2	124.0	6	0.000
	Medium	53	32	33	41	159	61.2			
	Tall	11	0	32	21	64	24.6			
	**Total**	**65**	**65**	**65**	**65**	**260**	**100**			
**Panicle compactness**	Loose	33	1	38	17	89	34.2	76.8	9	0.000
	Semi-loose	4	18	7	12	41	15.8			
	Semi-compact	27	44	19	26	116	44.6			
	Compact	1	2	1	10	14	5.4			
	**Total**	**65**	**65**	**65**	**65**	**260**	**100**			
**Panicle length**	Short	0	0	1	1	2	0.8	7.3	6	0.294
	Medium	11	7	15	7	40	15.4			
	Long	54	58	49	57	218	83.8			
	**Total**	**65**	**65**	**65**	**65**	**260**	**100**			
**Grain size**	Small	15	1	8	2	26	10.0	63.0	6	0.000
	Medium	27	8	38	29	102	39.2			
	large	23	56	19	34	132	50.8			
	**Total**	**65**	**65**	**65**	**65**	**260**	**100**			
**Grain color**	White	64	64	65	64	257	98.8	5.0	6	0.542
	Red	1	1	0	0	2	0.8			
	Yellow	0	0	0	1	1	0.4			
	**Total**	**65**	**65**	**65**	**65**	**260**	**100**			
**Plant cycle**	Early	24	41	7	8	80	30.8	62.1	6	0.000
	Medium	38	24	57	57	176	67.7			
	Late	3	0	1	0	4	1.5			
	**Total**	**65**	**65**	**65**	**65**	**260**	**100**			

## 4 Discussion

The low representation of females in sorghum cultivation reflects their limited access to land. Corrective measures through an important land reform by the government of the Republic of Senegal are underway to facilitate women's access to this resource [20]. Socio-cultural considerations also limit women's access to land. Sorghum cultivation was, for a long time, considered an activity for men because of the hard work involved. Farmers in these areas cultivate a wide range of crops such as pearl millet, sorghum, maize, rice, fonio, groundnut, cowpea, cotton and sesame during the rainy season. Among these, groundnut, sorghum, pearl millet and maize were ranked as the main crops, respectively. The strategy of growing several crops has been considered as a way to improve resilience [21]. However, according to Traoré et al. [22], it may prevent farmers from focusing sufficiently on adequate production of one particular crop. The main alternative crops to sorghum are groundnut, sesame and cotton. The importance of groundnut and cotton in rotation with sorghum is known and fully documented [23]. Crop rotation is known to be beneficial in the management of soil fertility, crop pests, and soil physical structure. Sorghum was preferentially grown as a sole crop by farmers (97.3 %). The use of improved varieties is limited, as 71.5 % of farmers are still cultivating local landraces. Farmers consider these landraces as cultural heritages from their ancestors to be transmitted to future generations. This strong relationship between farmers and their local cultivars is due to the rusticity and the convenience of these varieties to their local uses and conditions of production [24]. Local landraces, however, are known for their low response to fertilizers and have low yield compared to improved varieties and hybrids [8]. Overall low sorghum yield (939 kg ha^−1^) is obtained in Senegal [25] compared to a potential of 7–12 tons ha^−1^ [9,26]. Nevertheless, Trouche et al. [24] indicated that in low fertile conditions, local landraces of guinea type yield better than improved caudatum type. Improved varieties were not used in Bagadadji and Missirah, because farmers are not aware of their availability. While in Malicounda and Sagna, farmers have access to improved varieties introduced by RESOPP (*Réseau des Organizations Paysannes et Pastorales du Sénégal*) and ASPRODEB (*Association Sénégalaise pour la promotion du développement à la Base*), respectively. These two farmer organizations are acting on the training and dissemination of new technologies generated from agricultural research.

One to six different varieties were planted at a time by farmers in many localities. Diverse combinations such as early cultivars/late cultivars; local landraces/improved varieties or early cultivars/late cultivars/improved varieties have been reported by farmers. This approach is a strategy for farmers to mitigate risks associated with the rainy season [24] and also showed that yield was not usually the main focus of farmers. At Bagadadji and Missirah farmers produced their own seeds by selecting in their field the best panicles that met their criteria. Farmers reported that they did not know any official seed production service acting in their localities. This explains their lack of awareness regarding improved varieties released by the research. At Malicounda and Sagna, however, farmers have easy access to foundation and certified seeds. This is due to the farmer organizations COPAM (*Cooperative des producteurs de Malicounda*) and COPROSA (*Cooperative des producteurs du Saloum*), the extension service ANCAR (*Agence Nationale de Conseil Agricole et Rural*) and the private sector being active in their localities.

Farmers reported that the planting period depended on the onset of the rainy season. At Malicounda, where the rainy season starts late, certain farmers planted their sorghum fields before its onset in order to take advantage of the rain. In Senegal, this practice was common in pearl millet. Kanfany et al. [27] reported that 82 % of pearl millet farmers in the groundnut basin of Senegal plant their fields before the onset of the rains. Nevertheless, such practice subjected sorghum to early drought stress due to the unreliability of the rainy season [28,29].

The main sorghum production constraints raised by farmers were *Striga hermonthica*, insects, low soil fertility, and land access. Nevertheless, this general ranking can hide specificities among the rural communities. For instance, at Malicounda, bird attacks followed by insects and animal intrusion, were ranked by farmers as the main constraints. While at Sagna, Striga, land access and lack of rain were respectively ranked as the major constraints. In Senegal, for a long time, Striga was considered as only a constraint of pearl millet and not of sorghum. However, in this study Striga was ranked by farmers as the main constraint that affects considerably sorghum production in their localities. It was also shown in many Sub-Saharan African and Asian countries as one of the major constraints affecting sorghum cultivation and can be responsible for up to 90 % of yield loss [30]. Declining soil fertility and the overuse of their land could explain the emergence of Striga. According to Ramsom [31], the presence of Striga can be associated with low soil fertility, harsh environment, and high cultural intensity. Conversely, diseases were not ranked by farmers among the major constraints. Though, in Senegal, several reports of ISRA have identified grain mold and long smut as the most important diseases on improved sorghum varieties [32]. The low impact of diseases can be explained mainly by the predominant use of local landraces known for their high adaptation to local climate [24]. However, in Malicounda where improved varieties are mostly grown, the low pressure of diseases could be explained by the delay in the time of planting. In fact, farmers plant their fields late in August such that the grain filling and maturity periods occur after the end of the rains and escape mold infestation. This method is efficient in controlling diseases such as grain mold and anthracnose [33,34]. In addition, as most of the farmers of this locality are seed producers and members of the producer cooperative of Malicounda affiliated to RESOPP, they regularly get training on good agronomic practices. However, the delay in the time of planting to avoid grain mold has given rise to other constraints such as bird attacks and terminal drought which were ranked as the main sorghum production constraints by farmers at Malicounda. The ranking of main sorghum diseases by farmers has revealed that damping-off was the most important disease followed by grain mold and long smut. In fact, during the rainy season of 2013, most of the farmers interviewed in the rural communities of Sagna and Malicounda reported having a serious problem of seed germination and attributed this to damping-off. According to Chantereau et al. [35] damping-off of sorghum caused principally by *Pythium* ssp., *Fusarium* spp.*, Aspergillus* spp.*, Rhizoctonia* spp.*, and Phoma* spp, is the primary cause of non-germination of sorghum*.* However, grain mold could be also indicated in the decreased of germination and seedling vigor observed by farmers. Several studies showed that grain mold was associated with losses in seed mass, grain density, seed vigor, and seed germination [34,[36], [37], [38]]. Moreover, of the 260 farmers interviewed, 71 % reported having identified the disease on their field. The discoloration on the grain surface was the most important criteria used by farmers to identify the disease [39]. Farmers were aware that grain mold severity increased with rainfall at maturity, high moisture, and feeding insect attacks. According to Tonapi et al. [40], temperatures from 20 to 28°Cand relative humidity levels from 95 to 98%increased grain mold severity in most sorghum genotypes.

This study indicated that the majority of farmers preferred medium-height cultivars. These types of plant could be harvested without cutting them down, provide much fodder for animals and are suited for building houses. They also reported that tall sorghum cultivars (>300 cm) need to be cut down before harvesting and during this process many grains are lost. Tall cultivars are also late maturing and are usually subjected to terminal drought, bird attacks and animal intrusion. For these reasons, farmers from Malicounda opted for short plant varieties (<150 cm) that can be harvested without cutting them down. In addition, with the high pressure of birds in their locality, short types facilitate the management of bird attacks because they allow farmers to have a general view of the whole field.

Semi-compact and/or loose panicles were preferred by farmers. According to the farmers, compact panicles are reservoirs of insect pests and diseases that can seriously compromise the production. This is in agreement with Ratnadass et al. [41] who reported that feeding and oviposition of head-bugs on maturing sorghum grains result in severe losses, particularly on improved compact-headed types of the caudatum race. Similarly, Sharma et al. [42] reported that head-bug damage increases the severity of grain molds caused by species belonging to the genera *Fusarium, Curvularia, Phoma*, and *Alternaria*. Long sorghum panicles with medium or big grain size were also mentioned by farmers as important traits they desired in an improved sorghum variety. Several studies carried out in West and Central-Africa showed that yield, for farmers, is by far the most important objective to achieve in many improved crops such as rice [43], maize [44,45], sorghum [46], pearl millet [27,47] and cowpea [48]. However, in Africa yield is not usually the focus of farmers. Upon analyzing discussions with farmers during focus groups, it became evident that they placed greater importance on strategies that ensured them acceptable yield. Their mitigation strategies center around the utilization of up to three sorghum varieties, each with differing maturation cycles (early, medium, and late), aimed at averting potential production losses in the event of delayed or early onset of the growing season. Socio-cultural practices are also to be taken into consideration. For grain color, almost the majority of the farmers interviewed preferred white grained sorghum type. According to them, white grained cultivars are more suited for their local dishes. Preferences for white grained varieties was also reported in maize in Burkina Faso [44] and in cowpea in Ghana [49]. Furthermore, this study reveals that farmers preferred cultivars with medium (100–120 days) and/or short-duration cycle (<100 days). For these farmers, matching the cycle of varieties with the end of the rainy season is very important for them. According to Barro-Kondombo et al. [50], the duration of the cycle, which is significantly linked to the climatic zone, should be the principal criteria for adaptation of a variety for local climate. This could explain why the majority of the farmers at Bagadadji and Missirah are still using local photoperiodic landraces dominated by the Guinea race type. According to Traoré et al. [22], it is because they are photoperiod sensitive that local ecotypes match their cycle with the end of the ongoing rainy season. In the same way, Clerget [51] argues that the aptitude of photoperiodic ecotypes to match their flowering with the end of the rainy season is an essential key to adapting agriculture in the savannas of West Africa. Grain mold is most severe in early or medium-duration cultivars that mature during the rainy season under warm and wet conditions [52]. In Senegal, the introduction of improved non-photoperiodic varieties dominated by the Caudatum race type in sorghum growing areas could be a big challenge for the breeding program. Indeed, most of the varieties released by the breeding program these past ten years and recommended for the humid zones (South of the country) were susceptible to grain mold.

## 5 Conclusion

This study facilitated the identification of major challenges impairing sorghum cultivation in Senegal, namely, Striga, insect, low soil fertility, land access, and drought. The most important diseases are damping-off, grain mold and long smut. This study also shows that farmers are familiar with sorghum grain molds and have a good understanding of this disease. The results indicated that farmers' varietal trait preferences for a new improved sorghum variety are medium to short cycle, medium plant height, big and open or semi-compact panicle, big and white grain that are suitable for local dishes. In addition to these plant characteristics, resistant/tolerant to biotic and abiotic stresses such as Striga, insect, low soil fertility, drought and diseases were also needed by farmers in an improved sorghum variety. The results of this study also indicated that the sorghum cropping system is dominated by male farmers and local landraces. Improved varieties are mainly grown at Malicounda zone. Fertilizer and pesticide are used less by a few farmers. The findings from this study could be use by the sorghum breeding programs to update breeding product profile in order to better facilitate improved variety adoption in Senegal and other African countries. Further studies are also needed to analyze the sorghum value chain to better understanding the market demand, identifying and prioritizing key end-users’ requirements.

## Funding

The authors would like to thank the West-Africa Agricultural Productivity Program (WAAPP, Senegal) that partially funded this study and provided full scholarship to the first author. The authors also greatly appreciate the support of the American People provided to the Feed the Future Innovation Lab for Collaborative Research on Sorghum and Millet (SMIL) through the 10.13039/100000200United States Agency for International Development (USAID). The contents are the sole responsibility of the authors and do not necessarily reflect the views of USAID or the United States Government. Program activities are funded by the 10.13039/100000200United States Agency for International Development (10.13039/100000200USAID) under Cooperative Agreement No. AID-OAA-A-13-00047.

## Data availability

The data used to support the findings of this study are available from the corresponding author upon request.

## CRediT authorship contribution statement

**Cyril Diatta:** Writing – review & editing, Writing – original draft, Visualization, Formal analysis, Data curation, Conceptualization. **Thierry Klanvi Tovignan:** Writing – review & editing, Validation, Methodology, Formal analysis. **Bassirou Sine:** Writing – review & editing, Validation. **Beatrice Elohor Ifie:** Writing – review & editing, Validation, Supervision, Methodology. **Jacques Martin Faye:** Writing – review & editing. **Elisabeth Diatta-Holgate:** Writing – review & editing. **Fatou Anna Sylla:** Writing – review & editing. **Souleymane Bodian:** Writing – review & editing. **Ousmane Aidara:** Writing – review & editing. **Eric Yirenkyi Danquah:** Writing – review & editing, Validation, Supervision, Resources, Methodology, Investigation, Conceptualization. **Samuel Kwame Offei:** Writing – review & editing, Validation, Supervision, Resources, Methodology, Investigation, Conceptualization. **Ndiaga Cisse:** Writing – review & editing, Validation, Supervision, Resources, Project administration, Methodology, Investigation, Funding acquisition, Conceptualization.

## Declaration of competing interest

The authors declare that they have no known competing financial interests or personal relationships that could have appeared to influence the work reported in this paper.
