# Supplementary material for: Farmers’ production constraints, preferred varietal traits and perceptions on sorghum grain mold in Senegal
Source: Heliyon. 2024 Apr 26;10(9):e30221. doi: 10.1016/j.heliyon.2024.e30221 (PMC11070805; doi:10.1016/j.heliyon.2024.e30221)
Supplement: Multimedia component 1 [file mmc1.docx]

**PARTICIPATORY RURAL APPRAISALS QUESTIONNAIRE**

Questionnaire number: ……………..….………

Date of interview: ……….…/…………/……....

Name of interviewer: ……………….…...….…..

**IDENTIFICATION OF THE LOCALITY**

1. Agro-ecological zone: ………………..……

2. Name of the Rural Commune: ………….…

3. Name of the Village: ………..………..……

4. Geographic coordinate:

- Longitude: …………………………..
- Latitude: …………………………….
- Altitude: …………………………….

**IDENTIFICATION OF THE RESPONDENT**

5. Name of respondent: …………….………...

6. National ID Number: ………….….………..

7. Phone Number: ………………….…………

8. Age of Household head (in years)

- < 25
- 25-50
- 51-75
- > 75

9. Sex of household head

- Male
- Female

10. Ethnic group of household head

- Bambara
- Diakhanke
- Foulany
- Manding
- Nar
- Sarakhole
- Serrere
- Soninke
- Toucouleur
- Wolof

11. Marital status

- Single
- Married
- Divorced
- Widowed

12. Number of child

- No child
- 1-5
- 6-10
- 11-15
- 15

13. Level of education

- Illiterate
- Adult literacy
- Koranic study
- Primary
- Secondary
- Tertiary

14. Are you the household head?

- Yes
- No

15. Is farming your main sources of income?

- Yes
- No

16. Do you have other sources of income?

- Yes
- No

17. Are you a member of any farmers’ association?

- Yes
- No

**CROPPING SYSTEM**

18. Which are the major crops you grow?

- Groundnut
- Sorghum
- Pearl Millet
- Maize
- Cotton
- Rice
- Cowpea
- Sesame
- Fonio
- Others (specify)

19. Can you rank these crops by order of importance?

20. Do you grow sorghum?

21. Which type of sorghum varieties you grow?

- Landraces
- Improved
- Both landraces and improved

22. Can you list the main landraces you grow?

23. Where do you get the seeds of these landraces?

- Own seed
- NGOs
- Cooperative
- Market
- Relatives
- Others (specify)

24. What do you think about the seed quality?

- Good
- Medium
- Bad

25. Which of these improved varieties you grow?

- CE145-66
- CE151-262
- CE180-33
- CE196-7-2-1
- Darou
- F2-20
- Faourou
- Nganda
- Nguinthe
- Others (specify)

26. Where do you get the seeds of these varieties?

- Own seed
- Market
- NGOs
- Cooperative
- Others (specify)

27. What do you think about the seed quality?

- Good
- Medium
- Bad

28. How many sorghum varieties you grow per coping year?

- One
- Two
- Three
- Four
- Five
- Six

29. What are the alternate crop of sorghum in rotation?

- Groundnut
- Cowpea
- Cotton
- Sesame
- Maze
- Pearl millet
- Sorghum
- Fallow
- Others

30. How many field do you have?

- one
- Two-Four
- Five -Seven
- Eight -Ten
- More than ten

31. What is the size of your sorghum field?

- < 1 ha
- 1 - 2 ha
- 3 - 4 ha
- 5 - 6 ha
- > 6 ha

32. How do you plough your sorghum field?

- Tractor
- Donkey
- Horse
- Cow
- Donkey &Horse
- Horse & Cow
- Donkey & Cow
- Horse; Cow & Donkey

33. Do you grow sorghum in pure stands?

- Pure stands (sole)
- Mixed (intercropping)

33. Which period are you planting sorghum field?

- Before the rain
- Mid-June
- to Mid-July
- Mid-July to
- End-July
- Start-August to
- Mid-August
- Mid-August to End-August

34. Which period are you harvesting your sorghum field?

- Mid-October to Mid-November
- Mid-November to Mid-December
- Mid-December to Mid-January

35. Where you dry your harvest?

- Field
- House

36. How long you dry your harvest?

- Less a week
- one week
- Two weeks
- Three weeks
- one Month
- More a Month

37. Where you store your harvest?

- Warehouse
- Inside the house
- Hut near house
- Roof of the house

38. How do you store your sorghum seeds?

- Grain
- Bulk of panicle
- Panicle tied in bundle

**MANAGEMENT OF SORGHUM FIELDS**

39. Do you apply fertilizer?

- Yes
- No

40. Which type of fertilizer you apply?

- Organic
- Mineral

41. What is you appreciation about the price?

- Affordable
- Moderate
- Expensive

42. Do you apply pesticides?

- Yes
- No

43. Which type of pesticides you apply?

- Fungicide
- Herbicide
- Insecticide
- Nematicide
- Others (specify)

44. What is you appreciation about the price?

- Affordable
- Moderate
- Expensive

45. Where do you store your pesticides?

- Field
- House
- Store room

**USES OF SORGHUM**

46. What are your uses of sorghum grains?

- Household consumption
- Animal consumption
- Sale

47. What are your uses of sorghum straws?

- Building material
- Forage
- sale

**SORGHUM PRODUCTION CONSTRAINTS**

48. What are your major sorghum production constraints?

- Agricultural input
- Animal rambling
- Birds
- Cost of seeds
- Diseases
- Drought
- Insects
- Land access
- Poor soil fertility
- Striga
- Others (specify)

49. Can you rank these constraints by order of importance?

50. How do you manage these constraints?

51. Which of these diseases you encountered in your field?

- Long smut
- Grain mold
- Damping-off
- Ergot
- Foliar diseases
- Stem diseases
- Other diseases (specify)

52. Can you rank these diseases by order of importance?

53. How do you manage these diseases?

**FARMERS PERCEPTIONS’ ON SORGHUM GRAIN MOLD**

54. Do you know sorghum grain mold disease?

- Yes
- No

55. What name do you call this disease?

56. How do you recognize grain mold symptoms?

- Black color on the grain surface
- White color on the grain surface
- Pink color on the grain surface
- Others (specify)

57. What do you think causes grain mold?

- Rain at maturity
- Feeding insect
- Bad drying
- Early harvest
- Grain mold
- High moisture
- Dew
- Unknown
- Other (specify)

58. How do you try to control grain mold?

59. Do you face with any seed germination problem after planting?

- Yes
- No

60. What are the reasons of the non-germination of your seeds?

- Insects
- Seed quality,
- Lack of rain
- Seed quality
- Lack of rain
- Birds
- Bad grasses
- Low soil fertility
- No idea
- Others (specify)

**FARMERS’ PREFERRED TRAITS**

61. What type of plant you like?

- Tan
- Anthocyanin

62. What are your reasons?

63. Which plant height you prefer?

- Short (<150 cm)
- Medium (150-225 cm)
- Long (> 226 cm)

64. What are your reasons?

65. Which form of panicle you prefer?

- Loose
- Semi-loose
- Semi-compact
- compact

66. What are your reasons?

67. Which panicle size you prefer?

- Short (< 20 cm)
- Medium (21-30 cm)
- Long (> 30cm)

68. What are your reasons?

69. Which grain size you prefer?

- Small
- Medium
- Large

70. What are your reasons?

71. Which grain color you prefer?

- White
- Yellow
- Red
- Other (precise)

72. What are your reasons?

73. Which plant cycle you prefer?

- Early (<95 days)
- Medium (95-120 days)
- Late (>120 days)

74. What are your reasons?
