# Supplementary material for: Farmers’ production constraints, preferred varietal traits and perceptions on sorghum grain mold in Senegal
Source: Heliyon. 2024 Apr 26;10(9):e30221. doi: 10.1016/j.heliyon.2024.e30221 (PMC11070805; doi:10.1016/j.heliyon.2024.e30221)
Supplement: Multimedia component 2 [file mmc2.docx]

**Supplementary data**

**Table S1.** Number of improved and local sorghum varieties grown or lost by farmers in each rural community.

| **Type** | **Varieties** | **Bagadadji** | **Malicounda** | **Missirah** | **Sagna** |
| --- | --- | --- | --- | --- | --- |
| **Local varieties** |  |  |  |  |  |
|  | Anten-bi |  |  |  |  |
|  | Bassi |  |  |  |  |
|  | Bayan |  |  |  |  |
|  | Boroom Thiouck bouhonk |  |  |  |  |
|  | Boroom Thiouck bounioul |  |  |  |  |
|  | Bouda |  |  |  |  |
|  | Congossane |  |  |  |  |
|  | Dielani |  |  |  |  |
|  | Fella |  |  |  |  |
|  | Gadieum soukar |  |  |  |  |
|  | Kande dje* |  |  |  |  |
|  | Kande Woulema |  |  |  |  |
|  | Kintiry* |  |  |  |  |
|  | Kinto djema* |  |  |  |  |
|  | Kinto Woule* |  |  |  |  |
|  | Koumpa |  |  |  |  |
|  | Mbadar Cimakhan* |  |  |  |  |
|  | Mbayery danery |  |  |  |  |
|  | Mbayery Mbodery |  |  |  |  |
|  | Mbayery Mobal |  |  |  |  |
|  | Bassi Mbodiene |  |  |  |  |
|  | Nianikel |  |  |  |  |
|  | Nianikou |  |  |  |  |
|  | Niany Kinty* |  |  |  |  |
|  | Niodje |  |  |  |  |
|  | Niodje kounkounfi |  |  |  |  |
|  | Niodje kounkounwoule |  |  |  |  |
|  | Samba Diabo* |  |  |  |  |
|  | Teigne |  |  |  |  |
|  | Tellanior |  |  |  |  |
|  | Thiamery |  |  |  |  |
|  | Yabe* |  |  |  |  |
|  | Yamar* |  |  |  |  |
| **Research varieties** |  |  |  |  |  |
|  | CE151-262 |  |  |  |  |
|  | CE180-33 |  |  |  |  |
|  | Darou (ISRA-S-622B) |  |  |  |  |
|  | Faourou (ISRA-S-621B) |  |  |  |  |
|  | Nguinthe (ISRA-S-621A) |  |  |  |  |
|  | Nganda (ISRA-S-622A) |  |  |  |  |

- *variety grown in the rural communities, * = Variety abandoned by farmers*

**Table S2.** Reasons of sorghum seeds low germination rate in farmers’ fields

| **Trait** | **Rural communities** | | | | **Total** | **Percent** | **Chi-squares** | | |
| --- | --- | --- | --- | --- | --- | --- | --- | --- | --- |
|  | **Bagadadji** | **Malicounda** | **Missirah** | **Sagna** |  |  | **Value** | **Df** | **P.Value** |
| Insects | 1 | 10 | 8 | 11 | 30 | 12.8 | 123.7 | 51 | 0.0001 |
| Seed quality, and lack of rain | 6 | 5 | 3 | 3 | 17 | 7.3 |  |  |  |
| Lack of rain | 12 | 5 | 3 | 4 | 24 | 10.3 |  |  |  |
| Seed quality | 9 | 20 | 4 | 19 | 52 | 22.2 |  |  |  |
| Grain mold | 2 | 0 | 0 | 0 | 2 | 0.9 |  |  |  |
| Lack of rain, and insects | 4 | 4 | 5 | 12 | 25 | 10.7 |  |  |  |
| Seed quality, lack of rain and insects | 1 | 1 | 1 | 5 | 8 | 3.4 |  |  |  |
| Seed quality, and insects | 3 | 6 | 1 | 7 | 17 | 7.3 |  |  |  |
| Insects and birds | 3 | 1 | 1 | 0 | 5 | 2.1 |  |  |  |
| Birds, and lack of rain | 0 | 2 | 2 | 0 | 4 | 1.7 |  |  |  |
| Bad grasses | 0 | 2 | 1 | 0 | 3 | 1.3 |  |  |  |
| Lack of rain, and low soil fertility | 1 | 3 | 6 | 0 | 10 | 4.3 |  |  |  |
| Low soil fertility | 6 | 2 | 17 | 0 | 25 | 10.7 |  |  |  |
| Seed quality, and low soil fertility | 1 | 0 | 0 | 0 | 1 | 0.4 |  |  |  |
| Seed quality, insects and birds | 1 | 0 | 0 | 0 | 1 | 0.4 |  |  |  |
| Birds, lack of rain and insects | 1 | 0 | 0 | 0 | 1 | 0.4 |  |  |  |
| Low soil fertility, and insects | 1 | 0 | 0 | 0 | 1 | 0.4 |  |  |  |
| No idea | 3 | 1 | 4 | 0 | 2 | 0.9 |  |  |  |
| **Total** | **55** | **62** | **56** | **61** | **234** | **100.0** |  |  |  |
